# Mild Nutrient Starvation Triggers the Development of a Small-Cell Survival Morphotype in Mycobacteria

**DOI:** 10.3389/fmicb.2016.00947

**Published:** 2016-06-16

**Authors:** Mu-Lu Wu, Martin Gengenbacher, Thomas Dick

**Affiliations:** ^1^Antibacterial Drug Discovery Laboratory, Department of Microbiology and Immunology, Yong Loo Lin School of Medicine, National University of SingaporeSingapore, Singapore; ^2^Tuberculosis Research Laboratory, Department of Microbiology and Immunology, Yong Loo Lin School of Medicine, National University of SingaporeSingapore, Singapore

**Keywords:** bacterial differentiation, mycobacteria, quiescence, starvation, non-replicating bacteria, *Mycobacterium smegmatis*

## Abstract

Mycobacteria, generally believed to be non-sporulating, are well known to survive shock starvation in saline for extended periods of time in a non-replicating state without any apparent morphological changes. Here, we uncover that mycobacteria can undergo cellular differentiation by exposing *Mycobacterium smegmatis* to *mild* starvation conditions. Traces of various carbon sources in saline triggered the development of a novel small resting cell (SMRC) morphotype. Development of SMRCs could also be observed for other mycobacteria, suggesting evolutionary conservation of this differentiation pathway. Fluorescence microscopic analyses showed that development of SMRCs progresses via septated, multi-nucleoided cell intermediates, which divide to generate mono-nucleoided SMRCs. Intriguingly, saline shock-starved large resting cells (LARCs), which did not show cell size or surface changes when observed by scanning electron microscopy, remodeled their internal structure to septated, multi-nucleoided cells, similar to the intermediates seen during differentiation to SMRCs. Our results suggest that mycobacteria harbor a starvation-induced differentiation program in which at first septated, multi-nucleoided cells are generated. Under zero-nutrient conditions bacteria terminate development at this stage as LARCs. In the presence of traces of a carbon source, these multi-nucleoided cells continue differentiation into mono-nucleoided SMRCs. Both SMRCs and LARCs exhibited extreme antibiotic tolerance. SMRCs showed increased long-term starvation survival, which was associated with the presence of lipid inclusion bodies.

## Introduction

Bacteria in their terrestrial, aquatic or host environments constantly encounter nutrient deprivation and spend most of their life in non-growing resting states. Exponential growth under rich nutrient conditions is rare outside the laboratory (Matin et al., 1989; Rittershaus et al., 2013; Lenaerts et al., 2015). To endure these unfavorable conditions, survival responses have evolved into elaborate schemes of either physiological adaptation accompanied by distinct morphological changes or physiological adaptation only (Roszak and Colwell, 1987). Bacteria employing the former strategy are categorized as ‘differentiating’ or ‘sporulating’ bacteria, such as the endospore-forming *Bacillus subtilis* or the exospore-forming filamentous *Streptomyces coelicolor.* Whereas endospore formation starts with an asymmetric cell division followed by engulfment of the smaller pre-spore by the bigger ‘mother’ cell, exospore formation is initiated by septation of multi-nucleoided hyphae to form chains of mono-nucleoided pre-spore compartments. An example of non-differentiating bacteria are mycobacteria which belong – like *Streptomyces* – to the phylum Actinobacteria. Mycobacteria are capable of retaining viability when starved in saline for extended periods of time by entering a non-replicating resting state without any apparent morphological differentiation (Nyka, 1974; Gengenbacher et al., 2010). The saline starvation culture model for mycobacteria, established by Loebel a century ago (Loebel et al., 1933a,b), has witnessed a recent renaissance because the non-growing bacilli in this model display phenotypic drug resistance. Thus the ‘Loebel bacilli’ are regarded as persister bacilli and may represent a bacterial subpopulation in the host responsible for the difficulties in eradicating infections with antibiotics (Betts et al., 2002; Xie et al., 2005).

Whether morphologically differentiating bacteria execute their genetic program or not depends on the very specific culture conditions. For instance *B. subtilis* does not undergo endospore formation under all starvation conditions. As endosporulation is a prolonged (7 h compared to a generation time of 40 min) and energy-consuming process, the organism only commits to this developmental starvation-survival pathway when certain nutrients are present in the environment to allow completion of the process. *B. subtilis* does not enter the sporulation program under shock-starvation conditions (Hageman et al., 1984).

Based on this *Bacillus* behavior, we hypothesized that mycobacteria may harbor hidden morphological differentiation programs that could be uncovered by adding traces of nutrients into the standard saline starvation solution. We employed the mycobacterial model organism *Mycobacterium smegmatis* and subjected it to saline (shock) starvation or to gentle starvation in saline containing traces of carbon sources. The bacillus was able to survive medium term under both conditions. Shock-starved log-phase bacilli appeared to simply ‘freeze’ when encountering saline, i.e., they did not change size or shape in an apparent manner. In contrast, gently starved bacilli developed into a novel small-cell morphotype, which displayed increased long-term starvation survival. Here, we describe the cell biological and physiological characterization of this new morphological differentiation pathway in *Mycobacterium* spp.

## Materials and Methods

### Chemicals

Moxifloxacin, isoniazid, ethambutol, linezolid, clarithromycin, rifampicin, tyloxapol, and Tween80 were purchased from Sigma–Aldrich. Middlebrook 7H9 and Middlebrook 7H10 were purchased from Becton Dickinson. PBS was purchased from Invitrogen, Life Technologies (14080055).

### Bacterial Strains, Media, and Culture Conditions

*Mycobacterium smegmatis* mc^2^ 155 (ATCC 700084), *M. fortuitum* (ATCC 6841) and *M. peregrinum* (ATCC 23001) were grown at 37°C with agitation in Middlebrook 7H9 broth supplemented with 0.5% bovine albumin, 0.2% glucose, 0.085% NaCl, 0.5% glycerol, 0.0003% catalase, and 0.05% Tween80. For nutrient-starvation experiments, log-phase cultures with an optical density (OD) at 600 nm (OD_600_) of 0.5 were harvested by centrifugation (3200 rpm, 10 min, 25°C). After washing three times with PBS-0.025% Tween80 or PBS-0.025% Tyloxapol, the cultures were diluted to a final OD_600_ of 0.10–0.15. A volume of 50 ml of this suspension was transferred into a 1 liter roller bottle (Corning, COR430195) and starved for 14 days (or up to 6 months), with rolling at 2 rpm at 37°C. To verify that the small cells were not specific to Tween80 starvation, 0.005% of glucose, glycerol, or sodium acetate was used to replace Tween80. For all CFU determinations, appropriate dilutions of cultures were plated on Middlebrook 7H10 agar plates supplemented with 0.5% bovine albumin, 0.2% glucose, 0.085% NaCl, 0.5% glycerol, 0.0003% catalase, and 0.006% oleic acid. In regrowth experiments, 14-day-old cultures were spun down, re-inoculated into 7H9 broth to ∼OD_600_ 0.1 and incubated in T75 flasks with agitation at 15 rpm. For long-term starvation incubation experiments, cultures were topped up with sterile distilled water once a month to compensate for evaporation.

### Acid-Fast Staining and Light Microscopy

Acid-fast staining was carried out using a TB stain kit (BD, 212520) according to manufacturer’s instructions and observed under a light microscope (Olympus BX60, brightfield).

### Scanning Electron Microscopy

Fourteen-day-old starved cells and log-phase cultures were collected and fixed with 4% glutaraldehyde in 50 mM Tris/HCl pH7.2 buffer at 4°C overnight. After washing, pre-fixed samples were post-fixed in 1% OsO_4_ reagent for 1 h, followed by dehydration in gradually increasing concentration of ethanol up to 100%. Next, dehydrated samples were transferred to critical point dryer for infiltration with CO_2_. After mounting the specimen on scanning electron microscopy stubs, samples were sputter-coated with a thin layer of gold and examined using JEOL 5600 Scanning Electron Microscopy.

### FM4-64, DAPI, Nile Red Staining, and Fluorescence Microscopy

For membrane and DNA staining, samples collected at different time points were first fixed in 2% paraformaldehyde in PBS for 30 min and stained as previously described (Maloney et al., 2009). Briefly, fixed cultures were stained with the membrane dye FM4-64 (Molecular Probes, T3166) at a final concentration of 1 μg/ml for 1 h. Cells were harvested, stained with DAPI (Molecular Probes, D1306) at 10 μg/ml for 10 min, and then mounted to slides and visualized under either an epifluorescence microscope (Olympus BX60) or a confocal microscope (Olympus FV 1000 with TIRF microscopy). For the epifluorescence microscope, DAPI and FM4-64 stains were observed under U-M61002 and U-MWIG filters, respectively. For the confocal microscope, a filter set (Ex 528–553 nm/Em 600–660 nm) was used for FM4-64 and a standard DAPI filter set (Ex 325–375 nm/Em 435–485 nm) was used for DAPI-stained cells. Nile red (Sigma–Aldrich, N3013) staining was carried out similarly. Fixed samples were incubated at 5 μg/ml for 10 min, then visualized using confocal microscope at Ex 450–500 nm/Em > 528 nm. For fluorescence images, differential interference contrast (DIC) images were obtained simultaneously.

### Determination of Intracellular ATP Level

The BacTiter-Glo Microbial Cell Viability Assay (Promega) was employed to measure the ATP levels according to the manufacturer as described previously (Gengenbacher et al., 2010). A 25-μl volume of samples was mixed with an equal volume of the BacTiter-Glo reagent in 96-well white opaque Nunc plates. After 5 min of incubation in the dark, luminescence was measured (Tecan Infinite M200 Pro).

### Determination of Respiratory Rate

Bacilli were nutrient-starved for 14 days as described above. Aliquots of 2 ml (∼10^7^ CFU/ml), along with exponentially growing cultures (∼10^7^ CFU/ml) and 7H9 broth as controls, were transferred into clear round-bottom tubes and placed in an anaerobic jar. Oxygen was removed by AnaeroGen sachets (Oxoid). Methylene blue (1.5 μg/ml) was added into all tubes as an indicator of the oxygen status within the liquid phase. Color changes between starved cultures, exponentially growing cultures and broth reflect the relative oxygen consumption (Gengenbacher et al., 2010).

### Stress Exposure Experiments

Fourteen-day-old shock-starved cells, 14-day gently starved cells and exponentially growing bacilli were used for all stress exposure experiments. Cultures were diluted to approximately 10^7^ CFU/ml (unless stated otherwise) in the corresponding stress media. The survival of cells was monitored by CFU determination after treatment. Detergent stress was applied by addition of 0.5% SDS for 30 min. Acid stress was applied by addition of 6 M HCl to lower the pH to 1.5 for 20 min. For anaerobic stress, cultures were shifted to an anaerobic jar as described above and incubated for 2 weeks. For drug susceptibility test, all drugs were dissolved in 90% DMSO to prepare 5 mM working stock. The cidal activity of compounds against growing and non-replicating bacilli was determined by exposing an initial inoculum of approximately 10^6^ CFU/ml to 100 μM antibiotics in round-bottom tubes at 37°C and agitation at 200 rpm for 24 h.

## Results

### Mild Starvation Triggers the Formation of Small Resting Cells

**FIGURE 1:**
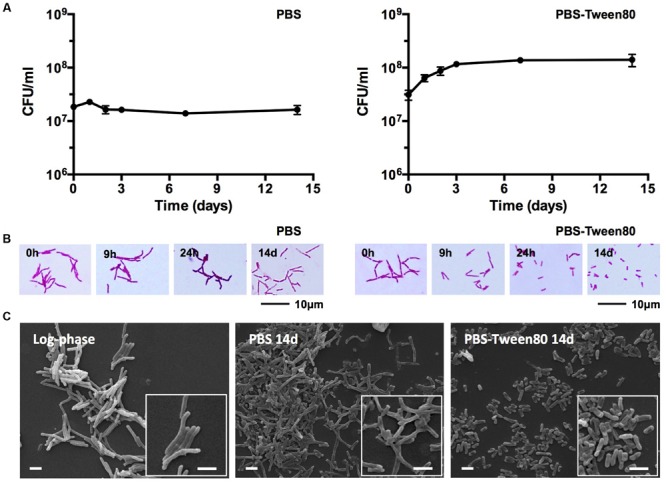
**Survival and cell shape of shock (PBS) and gently (PBS-Tween80) starved *M. smegmatis*. (A)** CFU of bacilli subjected to shock and gentle starvation over 14 days. Experiments were performed three times in triplicates and representative results are shown with means and standard deviations. **(B)** Light microscopy images of acid-fast stained bacilli exposed to shock and gentle starvation over 14 days. **(C)** Scanning electron microscopy images of log-phase and 14-day-old starved bacilli. White scale bars correspond to 2 μm. Representative fields are shown for **(B,C)**. Addition of Tween80 to 14-day-old PBS starved cultures did not result in the formation of small cells.

**FIGURE 2:**
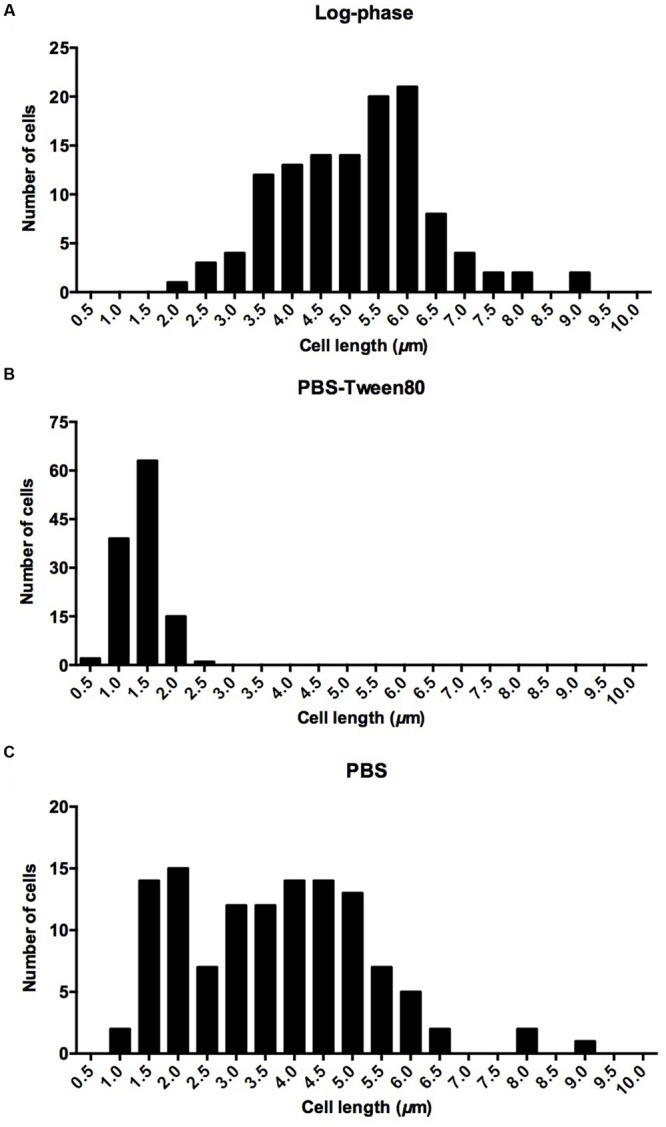
**Cell length distribution in **(A)** log-phase cultures, **(B)** 14-day-old cultures starved in PBS-Tween80, **(C)** 14-day-old cultures starved in PBS.** Eighty-five percentage of log phase cells had a length of 5.2 ± 0.9 μm. Ninety-four percentage of PBS-Tween80-starved cells had a length of 1.4 ± 0.3 μm. Seventy-five percentage of PBS-starved cells had a length of 4.1 ± 1.1 μm. One hundred and twenty cells were measured for each culture.

To determine mycobacterial survival under different levels of nutrient starvation we transferred exponentially growing *M. smegmatis* from rich 7H9 medium either to phosphate buffered saline (PBS) alone, or to PBS containing 0.025% Tween80, a fatty acid ester which is metabolized by mycobacteria (Tomioka, 1983; Lofthouse et al., 2013), and determined the number of viable cells by CFU measurement over time. **Figure 1A** shows that under both conditions the bacteria maintained full viability for 14 days, the duration of the experiment. In parallel, light microscopic inspection of acid-fast stained culture samples was carried out to detect any differences in cell morphology under the two different starvation culture conditions. The non-replicating PBS-starved bacteria did not show any apparent morphological changes (**Figure 1B**). In contrast, the long log-phase rods transferred to PBS-Tween80 solution developed into small cells within the first 2–3 days of starvation. **Figure 1C** shows scanning electron microscopy imaging of log-phase bacilli versus 14 days PBS- and PBS-Tween80-starved cells. Confirming the light microscopic observations, the PBS-treated cultures retained a shape and size similar to log-phase bacilli (4.1 ± 1.1 μm vs. 5.2 ± 0.9 μm, see **Figures 2A,C** for cell length distributions). In contrast, the gently starved bacilli were about threefold to fourfold shorter with an average length of 1.4 ± 0.3 μm (see **Figure 2B** for cell length distribution). Note that whereas exponentially growing and PBS-starved cultures exhibited some degree of heterogeneity in cell length, the PBS-Tween80-starved cultures showed a high degree of homogeneity: >94% of the cells were of the small-cell (1.4 μm) morphotype (**Figure 2B**). These results suggest that gentle nutrient starvation triggers the development of a new small-cell resting form in *M. smegmatis*.

To determine whether the development of small cells was specific to Tween80 as carbon source we replaced this fatty acid ester with traces of glucose, glycerol, or acetate, respectively. Supplementary Figure S1 depicts conversion of the cultures to the small cell morphotype for these other carbon sources, suggesting that the generation of the small-cell morphotype was independent of the nature of the carbon source.

To determine whether the gentle starvation-induced formation of small resting cells (SMRCs) is specific to *M. smegmatis*, we carried out starvation experiments with other mycobacteria. Supplementary Figure S2 shows that gentle starvation-induced small-cell formation could also be observed for *M. fortuitum* and *M. peregrinum*, suggesting that this starvation-induced differentiation response is a trait shared by other mycobacterial species.

Taken together, the comparative starvation experiments revealed that the non-differentiating mycobacteria actually can undergo morphological differentiation when exposed to mild starvation conditions: traces of various carbon sources in saline triggered the development of a novel SMRC morphotype. Formation of SMRCs could also be observed for other mycobacteria, suggesting a broader occurrence of this new differentiation pathway. Shock starvation did not result in any apparent morphological changes and the cultures showed structurally unaltered large resting cells (LARCs).

### Small Resting Cells Grow Out to Large Cells before Resuming Cell Division

**FIGURE 3:**
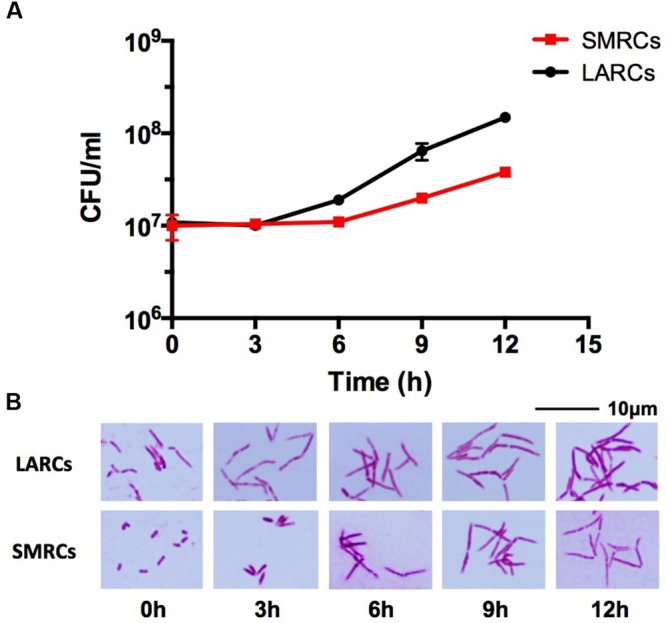
**Growth and cell shape of 14-day-old PBS-Tween80 (small resting cells, SMRCs) or PBS (large resting cells, LARCs) starved *M. smegmatis* after transfer to rich medium. (A)** CFU of starved bacilli after transfer to 7H9 medium over time. The experiment was performed three times in triplicates and a representative example is depicted showing means with standard deviations. **(B)** Light microscopic images of acid-fast stained bacilli sampled at indicated time points. Representative fields are shown.

Given that the 1.4 μm mini-rod SMRCs indeed represent a form of resting cell, we expected that SMRCs would grow out to log-phase-length bacilli before resuming multiplication. To determine whether SMRCs indeed exhibit this regrowth behavior we carried out regrowth experiments by transferring 14-day-old PBS-Tween80-starved *M. smegmatis* into rich 7H9 medium. **Figure 3** shows the results of CFU determination and microscopic observations over time. The regrowth culture exhibited a lag phase of 6 h without increase in CFUs (**Figure 3A**). During this lag phase all SMRCs converted into large cells (**Figure 3B**), and only after cell length extension was an increase in CFU observed. This result suggests that the SMRC morphotype itself is not multiplication proficient but rather represents a resting cell type, which needs to grow out to a ‘vegetative’ cell before resuming cell division. **Figure 3A** depicts that regrowth experiments of LARCs in rich broth also showed a somewhat shorter lag phase before CFU numbers increased. **Figure 3B** shows that no apparent cell size changes occurred during regrowth of LARCs.

### Small Resting Cells Develop via Septated, Multi-Nucleoided Cells

**FIGURE 4:**
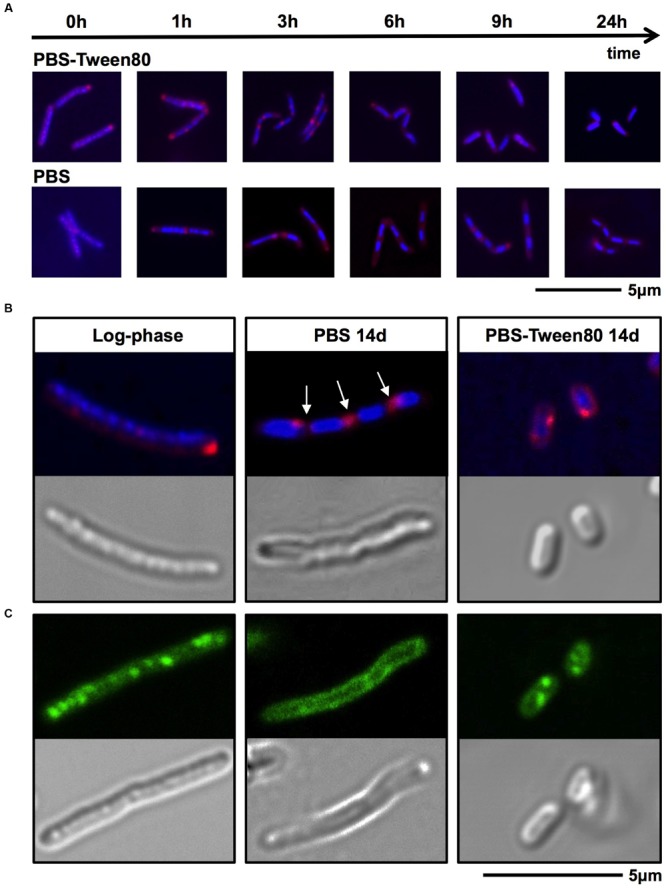
**DNA, membrane and fat body visualization of gently (PBS-Tween80) and shock (PBS) starved *M. smegmatis*. (A,B)** Log-phase cultures were subjected to gentle and shock starvation. Samples were collected over time and stained with DAPI (blue) to visualize DNA and FM4-64 (red) to visualize membranes. **(A)** Fluorescence microscopy imaging of samples taken during the first 24 h of starvation. **(B)** Confocal microscopy imaging of DAPI and FM4-64 stained log-phase and 14-day-old starved bacilli, and the corresponding DIC images. Arrows indicate apparent septa. **(C)** Confocal microscopy imaging of Nile red (green) stained log-phase bacilli and 14-day-old starved bacilli to visualize fat bodies. Corresponding DIC images are shown in the lower panel. Fields shown are representative.

To dissect the cellular mechanisms of SMRC formation from vegetative log-phase cells we carried out fluorescence microscopic analyses of *M. smegmatis* transferred to PBS-Tween80. DAPI and FM4-64 were used to visualize DNA or membrane, respectively. **Figure 4A** shows that a large fraction of log-phase cells had formed septated, di- and tetra-nucleoided cells during the first 6 h. After 24 h most of the culture consisted of a homogeneous population of 1.4-μm long mono-nucleoided small cells (see **Table 1** for quantification). These results suggest that SMRCs are generated in a two-step process: first log-phase cells differentiate into septated, multi-nucleoided cells, which then divide to generate separated daughter SMRCs. The expected corresponding slight increase in cell number during the initial phase of PBS-Tween80 starvation can actually be observed: as shown in **Figure 1A**, gentle starvation resulted in a threefold to fourfold increase in CFUs during the first 2–3 days of starvation after which CFUs stayed constant.

**Table 1:** Distribution of mono- and multi-nucleoided cells in PBS and PBS-Tween80 starved cultures over time.

Starvation condition	Time	No. of nucleoids per cell			
		1	2	3	4	*n*^a^
PBS	6 h	17%	66%	–	17%	53
	9 h	18%	40%	11%	31%	72
	14 days	24%	29%	5%	42%	38
PBS-Tween80	6 h	41%	46%	–	12%	41
	9 h	53%	30%	4%	13%	47
	14 days	95%	5%	–	–	50

*^a^Number of cells quantified in each sample.*

### Shock-Starved Large Resting Cells Remodel Their Interior to a Septated, Multi-nucleoided Cell Form

Surprisingly, fluorescence microscopic analysis of PBS shock-starved *M. smegmatis*, also revealed the formation of septated, di- and tetra-nucleoided cells within the first 6 h of starvation (**Figure 4A**) and 76% of 14-day-old cultures consisted of 4 μm long multi-nucleoided cells (**Figure 4B**, see **Table 1** for quantification). Thus, LARCs are in fact morphologically not simply growth-arrested log-phase cells as could have been concluded from light and scanning electron microscopic analyses (**Figure 1C**). Rather, the generation of LARCs involves an internal remodeling of cells to septated, multi-nucleoided forms. The finding that a septated, multi-nucleoided cell form represents on the one hand an apparently terminally differentiated cell form in the case of LARC development, and on the other hand an intermediate cell stage during SMRC development, suggests that both, SMRC and LARC development share a similar genetic program.

### Small Resting Cells, Similar to Large Resting Cells, Exhibit Reduced Metabolism, General Stress Resistance, and Extreme Antibiotic Tolerance

Reduced metabolic activity and elevated general stress resistance are hallmarks for starvation-induced resting states of all bacteria (Rittershaus et al., 2013). This has also been demonstrated previously for PBS (shock)-starved mycobacteria. Bacilli in this non-replicating state are characterized by a reduced intracellular ATP concentration, reduced oxygen consumption, and increased survival under various stress conditions including acidic pH, detergent, anaerobiosis as well as antibiotic stress (Loebel et al., 1933a,b; Smeulders et al., 1999; Betts et al., 2002; Xie et al., 2005; Gengenbacher et al., 2010; Sarathy et al., 2013). We interrogated whether these adaptations can also be observed for *M. smegmatis* LARCs and the new SMRC morphotype. To this end we subjected log-phase cells to PBS and PBS-Tween80 starvation and measured cellular ATP concentration, oxygen consumption, and survival under various stresses.

**FIGURE 5:**
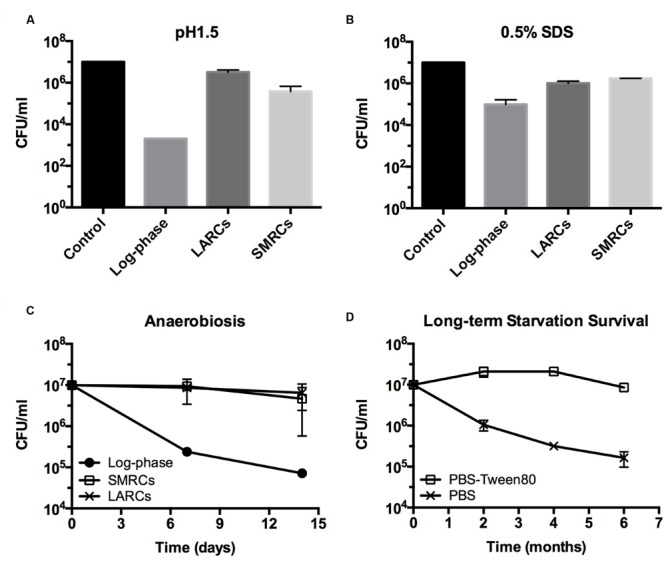
**Stress tolerance and long-term survival of shock (PBS) starved and gently (PBS-Tween80) starved *M. smegmatis*. (A,B)** CFU determinations after exposure of starved 14-day-old cultures to acid (pH 1.5, 20 min) or detergent (0.5% SDS, 30 min) stress. **(C)** Survival curves of starved cultures exposed to anaerobiosis using anaerobic jars. LARCs, 14-day-old PBS starved cultures. SMRCs, 14-day-old PBS-Tween80 starved cultures. Log-phase, log-phase cultures. **(D)** Long-term viability of log-phase bacilli exposed to PBS-Tween80 and PBS starvation. Data shown are means and standard deviations from three independent biological replicates.

Intracellular ATP dropped twofold from (2.86 ± 0.52) × 10^-18^ mol/CFU in log-phase cultures to (1.39 ± 0.04) × 10^-18^ mol/CFU in PBS-starved cultures. In PBS-Tween80-starved cultures ATP dropped ninefold to (3.16 ± 1.03) × 10^-19^ mol/CFU. Considering the threefold to fourfold smaller size of SMRCs compared to LARCs, this result suggests that both starved cell types exhibited a similar, about twofold reduced, intracellular ATP concentration. Supplementary Figure S3 depicts that SMRCs, similar to LARCs, showed reduced oxygen consumption when compared to log-phase bacilli. Furthermore, exposure of starved cultures to various stresses revealed that SMRCs, again similar to LARCs, displayed increased survival under acidic pH (**Figure 5A**), detergent exposure (**Figure 5B**), anaerobiosis (**Figure 5C**), and antibiotic stress (**Table 2**). Collectively, our data suggest that both SMRCs and LARCs have reduced their metabolism together with increased general stress tolerance, including extreme antibiotic tolerance.

**Table 2:** Drug susceptibility of 14-day-old PBS-Tween80 (SMRCs) and PBS (LARCs) starved cultures vs. susceptibility of log-phase cultures.

Drug^a^	Fold kill^b^		
	SMRCs	LARCs	Log-phase
Moxifloxacin	6.5	65	>50,000
Isoniazid	1	2.5	60
Ethambutol	1	3	20
Rifampicin	1	2.5	40
Linezolid	1	2.8	30
Clarithromycin	1	3	14

*Fold kill is shown after 1 day of exposure. ^a^All drugs were tested at 100 μM. ^b^Fold kill was calculated by comparing survival CFU with starting inoculum (∼10^*6*^ CFU/ml).*

### Small Resting Cells Contain Fat Storage Bodies and Show Prolonged Starvation Survival as Compared to Large Resting Cells

Mycobacteria growing in rich media accumulate intracellular fat bodies as storage material (Low et al., 2009). To determine how the different starvation conditions affect fat storage we carried out fluorescence microscopy of growing and starved cultures with Nile red stain to visualize lipid inclusions. As expected, log-phase *M. smegmatis* contained a large number of fat bodies (**Figure 4C**). No fat bodies were detectable in PBS shock-starved cells. In contrast, even after 14 days of starvation, SMRCs still retained some fat bodies (**Figure 4C**). Based on the observation that SMRCs retained lipid inclusions, we hypothesized that the SMRCs display better long-term starvation survival compared to LARCs. Data in **Figure 5D** confirm our prediction. SMRC cultures maintained an almost constant CFU concentration over 6 months of starvation, whereas LARC cultures showed a 65-fold drop in viable numbers over the same period of time. We conclude that SMRCs are better adapted to survive long-term starvation.

## Discussion

There are bacteria that form specialized, i.e., morphologically differentiated resting cells and non-sporulating bacteria that do not do so. The most widely appreciated bacterial non-growing survival form is best presented by the extremely resistant and persistent resting endospore of *B. subtilis* (McKenney et al., 2013). Recently, Ghosh et al. (2009) reported the discovery of endospores in ‘old’ cultures of *Mycobacterium bovis* BCG and *Mycobacterium marinum*, and described striking similarities to *B. subtilis* endospores regarding structure (spore coat), properties (spore staining, heat resistance), and chemistry (dipicolinic acid). This finding was rejected by Traag et al. (2010) who pointed out that mycobacteria lack critical orthologs of highly conserved endospore genes. Development of specific *Bacillus*-like endospores by mycobacteria thus remains controversial, though unlikely (Lamont et al., 2012; Wu and Dick, 2015). Certain bacteria do actually form other types of specialized resting cells, however, these resting cells are not endospore-like in regard to structure and extreme resistance, and are generated by different cellular mechanisms. ‘Exospores’ formed by the *Mycobacterium* spp. relatives *Streptomyces* spp. are an example. These resting cells are generated via septation and separation of multi-nucleoided hyphae (Scherr and Nguyen, 2009).

**FIGURE 6:**
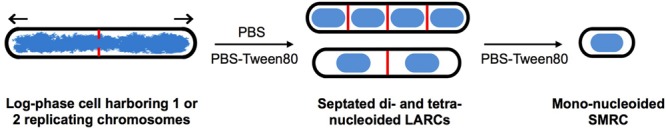
**Nutrient starvation-induced differentiation in *M. smegmatis*.** Model depicting starvation-induced differentiation of log-phase cells first into LARCs. Under zero-nutrient starvation (PBS), development stops here. In the presence of traces of a carbon source (PBS-Tween80), LARCs undergo cell division and separate into SMRCs. Blue: DNA, red: septa, black: cell envelope. Arrows indicate polar growth of log-phase cells.

Mycobacteria are very hardy and can for instance survive shock starvation in phosphate buffered saline in a non-replicating state without any apparent morphological differentiation (Sarathy et al., 2013). Here, we decided to have another look at this adaptation but with shifting the cone of the streetlight (Freedman, 2010): rather than shock-starving the bacilli in saline we added traces of a carbon source to the saline. This idea was inspired by the lesson learnt from the sporulation process in *B. subtilis*. Spore formation is triggered under starvation. However, *Bacillus* first confirms availability of carbon sources before committing to the energy consuming and rather lengthy endosporulation process (Stephens, 1998). Intriguingly, traces of a carbon source to *M. smegmatis* in saline, i.e., gently as opposed to shock-starving the organism, caused the formation of a small cell morphotype. In deviation from the standard log-phase cell division cycle, first septated, multi-nucleoided resting cells were formed. Subsequently, these cells underwent cell division and generated very short mini rods with increased long-term viability (**Figure 6**). Upon addition of rich medium, these SMRCs grew back to larger standard cells before commencement of the regular cell division cycle. We termed these specialized resting cells SMRCs, avoiding the word ‘spore’ to prevent any confusion with *Bacillus* endospores or *Streptomyces* exospores. Nevertheless, the superficial resemblance with exospore formation in *Streptomyces*, septation of multi-nucleoided cells followed by cell separation, is intriguing (Scherr and Nguyen, 2009). Development of SMRCs could also be observed for other mycobacteria, suggesting a broader presence of this new differentiation pathway in this genus. Surprisingly, saline shock-starved LARCs, which did not show any apparent cell changes in size or surface by scanning electron microscopy, underwent remodeling of their internal structure to the septated, multi-nucleoided cells occurring during differentiation to SMRCs.

Taken together, our observations suggest that mycobacteria harbor a starvation-induced differentiation program in which at first septated, multi-nucleoided cells are generated. Under zero-nutrient conditions the bacilli terminate development at this stage as LARCs. In the presence of traces of a carbon source, these multi-nucleoided cells continue differentiation: they complete cell division and separate into mono-nucleoided SMRCs (**Figure 6**).

What could be the underlying rationale for shock starvation-induced LARC, and gentle starvation-induced SMRC formation? And how is this related to canonical, log-phase cell division cycle? Cell division cycle analyses of *M. smegmatis* growing in rich medium revealed that the longest part (140 min) of the full generation time (180 min) is spent in the C-phase, i.e., chromosome replication. Moreover, a large fraction of those C-phase log cells contain two replicating chromosomes (Aldridge et al., 2012; Santi et al., 2013; Kieser and Rubin, 2014). This is consistent with the observed predominance of di- and tetra-nucleoided cells in LARCs and the LARC-like cell intermediates observed during SMRC development: upon sensing starvation the C-phase cells harboring one or two replicating chromosomes appear to complete their ongoing DNA syntheses, segregate their chromosomes and form septa, i.e., they form compartments containing a single compacted chromosome (**Figure 6**). Cells cultured in PBS stop at that stage as LARCs, likely because they run out of their intracellular stored carbon source (fat bodies) required for synthesis of the highly massive mycobacterial cell envelope [60% of dry weight in mycobacteria consists of cell envelop material, compared to 10% in *Escherichia coli* (Brennan and Goren, 1979; Brennan and Nikaido, 1995)]. In contrast, the septated multi-nucleoided cells in PBS-Tween80 appear to sense the traces of available carbon sources allowing the completion of cell envelope synthesis and physical separation (**Figure 6**). By increasing cell numbers, this step may enable the organism to spread to favorable environments. Furthermore, SMRCs are equipped with a longer half-life compared to LARCs, likely due their ability to maintain fat bodies as a form of energy storage. These two survival advantages of SMCRs over LARCs — higher number of ‘shots’ to land on spots with favorable microenvironments and longer half-life — may be the evolutionary rationale for favoring SMRC development taking place when the environment provides traces of carbon for the cells to complete cell envelope synthesis and cell separation.

Isolated reports have described various unusual small mycobacterial cell shapes *in vitro* and *in vivo* (Smeulders et al., 1999; Ojha et al., 2000; Berney and Cook, 2010; Shleeva et al., 2011; Wu and Dick, 2015), including the formation of small ovoid *M. smegmatis* cells observed in old, non-agitated nitrogen-limited minimal medium stationary phase cultures (Anuchin et al., 2009). Similar to SMRCs, these small ovoid shaped persister cells reported by Kaprelyants and colleagues showed low metabolic activity and increased antibiotic resistance. Interestingly, the ovoid starved cells are ‘viable but not culturable’ (Shleeva et al., 2004; Salina et al., 2010; Nazarova et al., 2011) and upon resuscitation, they exhibited a ‘budding’ or ‘germination-like’ regrowth pattern. These phenotypes were not observed for SMRCs. The differences between the small ovoid form persisters and SMRCs might be due to the specific culture conditions resulting in different types of resting cells via different mechanism.

The results of this study have several implications. The discovery of SMRCs demonstrates that the non-sporulating mycobacteria are in fact capable of undergoing morphological differentiation into resting cells. We also consider our findings of clinical relevance. Mycobacterial infections, including increasing incidences of diseases caused by non-tuberculous mycobacteria (NTM; Katoch, 2004; Griffith et al., 2007), are notoriously difficult to cure with chemotherapy (Jarand et al., 2011). The highly stress tolerant, phenotypically antibiotic-resistant and long-living SMRCs discovered in this study may contribute to this obstacle. Future work will identify the genetic program controlling this novel mycobacterial developmental process.

## Author Contributions

M-LW, MG, and TD conceived the project and designed the strategy. M-LW carried out the experiments. M-LW and TD analyzed the data and wrote the manuscript.

## Conflict of Interest Statement

The authors declare that the research was conducted in the absence of any commercial or financial relationships that could be construed as a potential conflict of interest.
