# Supplementary material for: Mild Nutrient Starvation Triggers the Development of a Small-Cell Survival Morphotype in Mycobacteria
Source: Front Microbiol. 2016 Jun 16;7:947. doi: 10.3389/fmicb.2016.00947 (PMC4909757; doi:10.3389/fmicb.2016.00947)
Supplement: Supplementary file 1 [file Data_Sheet_1.PDF]

Supplementary Material

Mild Nutrient Starvation Triggers the Development of a Small-Cell Survival Morphotype in Mycobacteria

Mu-Lu Wu[1], Martin Gengenbacher[2], Thomas Dick[1]*

*** Correspondence:** Thomas Dick: thomas_dick@nuhs.edu.sg

1 Supplementary Figures

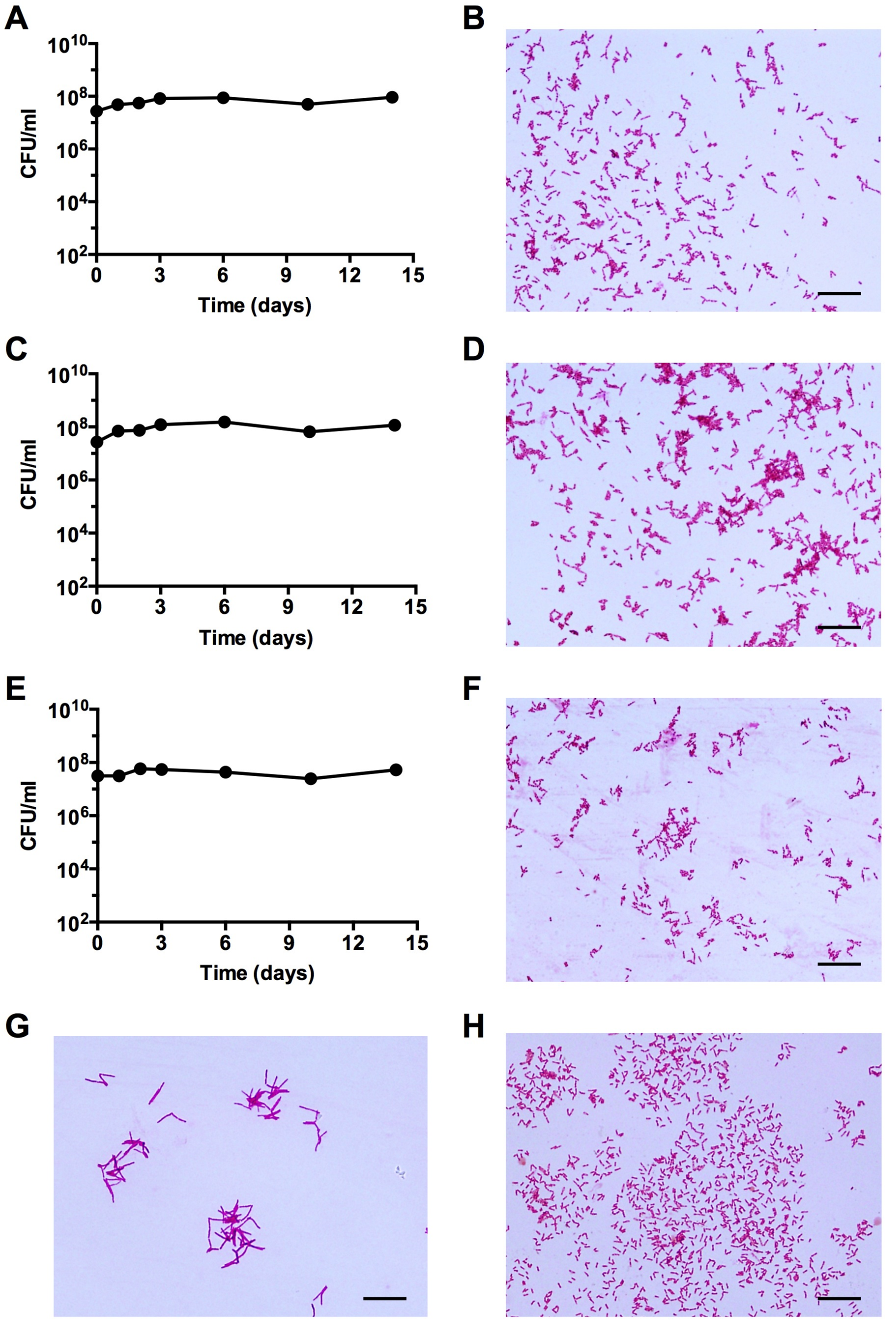

Supplementary Figure 1. Survival and cell shape of *M. smegmatis* starved in PBS containing traces of glucose, glycerol or acetate instead of Tween80. Tween80 was replaced by 0.005% glucose **(A-B)**, 0.005% glycerol **(C-D)** or 0.005% acetate **(E-F)**. **(A,C,E)**: CFU of cultures over 14 days (mean ± sd). **(B,D,F)**: Microscopic images of acid-fast stained 14-day-old cultures. **(G,H)**: Microscopic images of acid-fast stained log-phase **(G)** and 14-day-old PBS-Tween80 starved cultures **(H)** are shown as reference. Images shown are representative fields. Black scale bars on the figures correspond to 15 μm. Note: The specific carbon source concentrations chosen were the result of titration experiments: higher concentrations did not result in the formation of the small cell morphotype.

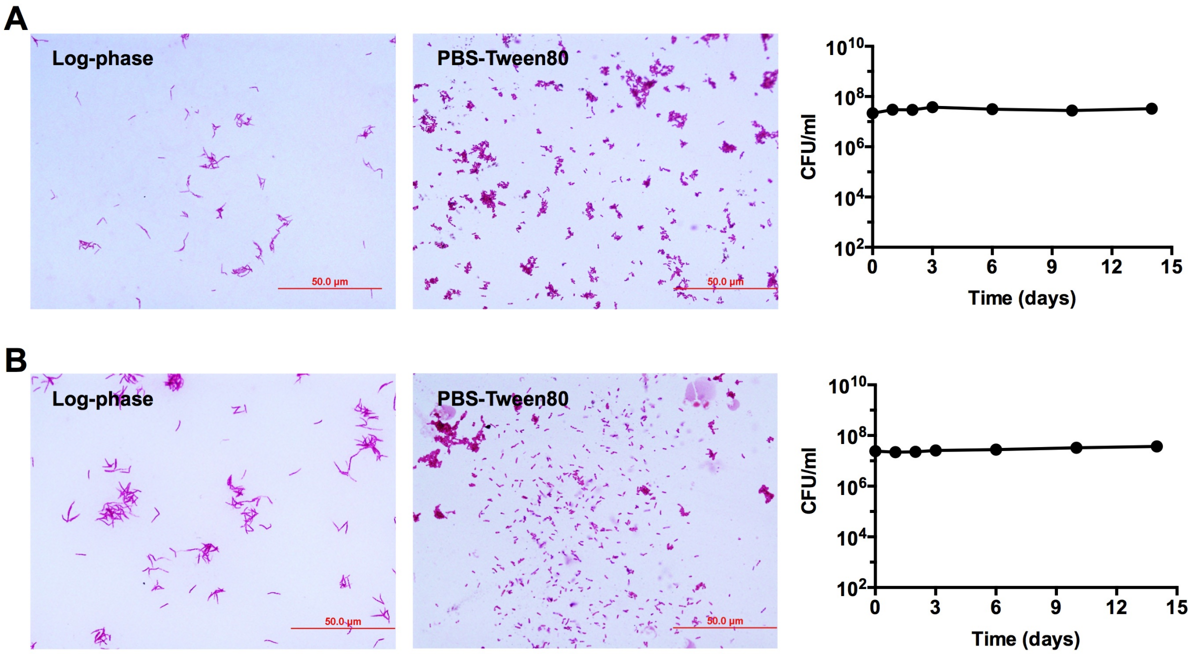

Supplementary Figure 2. Survival and cell shape of *M. fortuitum* (A) and *M. peregrinum* (B) starved in PBS-Tween80. Microscopic images shown are representative fields of acid-fast stained log-phase and starved 14-day-old PBS-Tween80 cultures. The CFU determinations were carried out three times and a representative example is shown (mean ± sd).

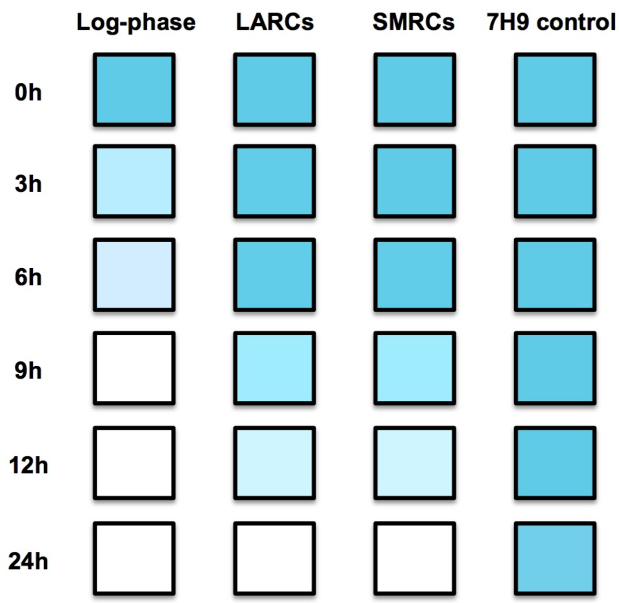

Supplementary Figure 3. Oxygen consumption of log-phase vs 14d old PBS or PBS-Tween80 starved cultures. 2 ml log-phase cultures in 7H9, 14d old shock starved PBS cultures and 14d old gently PBS-Tween80 starved cultures were transferred to an anaerobic jar after adding oxygen indicator methylene blue (0h). Decolorization over time, a qualitative measure of oxygen consumption, is shown. 7H9 medium without bacteria was used as negative control. Methylene blue decolorized rapidly in log phase culture samples by 9h. 14d old PBS and PBS-Tween80 cultures decolorized methylene blue after 24h, indicating reduced (and similar) oxygen consumption of both starved cells types. The fact that both starved cultures do consume oxygen, albeit at a reduced level, suggests that both, LARCs and SMRCs still maintain a low level metabolism.
